# Forecasting the long-term trend of COVID-19 epidemic using a dynamic model

**DOI:** 10.1038/s41598-020-78084-w

**Published:** 2020-12-03

**Authors:** Jichao Sun, Xi Chen, Ziheng Zhang, Shengzhang Lai, Bo Zhao, Hualuo Liu, Shuojia Wang, Wenjing Huan, Ruihui Zhao, Man Tat Alexander Ng, Yefeng Zheng

**Affiliations:** Jarvis Lab, Department of Medicine and Healthcare, Tencent Technology (Shenzhen) Company, Shenzhen, 518000 China

**Keywords:** Viral infection, Epidemiology, Experimental models of disease, Population screening, Risk factors

## Abstract

The current outbreak of coronavirus disease 2019 (COVID-19) has recently been declared as a pandemic and spread over 200 countries and territories. Forecasting the long-term trend of the COVID-19 epidemic can help health authorities determine the transmission characteristics of the virus and take appropriate prevention and control strategies beforehand. Previous studies that solely applied traditional epidemic models or machine learning models were subject to underfitting or overfitting problems. We propose a new model named Dynamic-Susceptible-Exposed-Infective-Quarantined (D-SEIQ), by making appropriate modifications of the Susceptible-Exposed-Infective-Recovered (SEIR) model and integrating machine learning based parameter optimization under epidemiological rational constraints. We used the model to predict the long-term reported cumulative numbers of COVID-19 cases in China from January 27, 2020. We evaluated our model on officially reported confirmed cases from three different regions in China, and the results proved the effectiveness of our model in terms of simulating and predicting the trend of the COVID-19 outbreak. In China-Excluding-Hubei area within 7 days after the first public report, our model successfully and accurately predicted the long trend up to 40 days and the exact date of the outbreak peak. The predicted cumulative number (12,506) by March 10, 2020, was only 3·8% different from the actual number (13,005). The parameters obtained by our model proved the effectiveness of prevention and intervention strategies on epidemic control in China. The prediction results for five other countries suggested the external validity of our model. The integrated approach of epidemic and machine learning models could accurately forecast the long-term trend of the COVID-19 outbreak. The model parameters also provided insights into the analysis of COVID-19 transmission and the effectiveness of interventions in China.

## Introduction

Coronavirus disease 2019 (COVID-19) is infectious pneumonia caused by severe acute respiratory syndrome coronavirus 2^1^. The disease was first reported in December 2019 in Wuhan city, the capital of Hubei province in China, and has since then spread across China and globally^2^. As of 19 August, a total of 22 million COVID-19 cases and 773,067 deaths have been reported in more than 200 countries and territories^3^. The World Health Organization (WHO) has declared the COVID-19 outbreak as a Public Health Emergency of International Concern and a pandemic recently^4^.

Forecasting the long-term trend of the epidemic can help health authorities determine the transmission characteristics of the virus and develop appropriate prevention and containment strategies beforehand. Recently, some researchers applied the traditional epidemic models like Susceptible-Exposed-Infective-Recovery (SEIR) or machine learning models like logistic regression to predict the trend of COVID-19^5,6^. To the best of our knowledge, most of those researches were performed retrospectively, or subject to overfitting or underfitting problems. The validity of the SEIR model depends on accurate estimation of virus transmission characteristics such as the basic reproduction number R_0_, incubation period, and infectious period. In a real scenario, those parameters are not easy to estimate. For example, Wu et al. made an estimation of the basic reproduction number using exported cases from China to abroad/overseas and estimated that 75,815 individuals had been infected in Wuhan as of 25 January^6^, which significantly overestimated the figure. On the other hand, due to insufficient training data and valid features, machine learning models were subject to overfitting, restricted to retrospective analysis, or only forecasting short-term trends^5,7–10^.

To address the aforementioned issues, we propose a novel model named Dynamic-Susceptible-Exposed-Infective-Quarantined (D-SEIQ), by making appropriate modifications of the SEIR model and integrating machine learning based parameter optimization under reasonable constraints. Our D-SEIQ model effectively improves the performance of long-term trend forecast for COVID-19 outbreak in China and five other countries. In addition, the model parameters, such as the dynamic reproduction number, could provide insights into the analysis of COVID-19 transmission characteristics and the effectiveness of interventions.

## Methods

### D-SEIQ model

The primary differences from our D-SEIQ model and SEIR model include (1) replacing recovered individuals $R$ with quarantined individuals $Q$, and (2) introducing time-dependent dynamics to the estimation of the effective reproduction number ${R}_{t}$.

**Figure 1:**
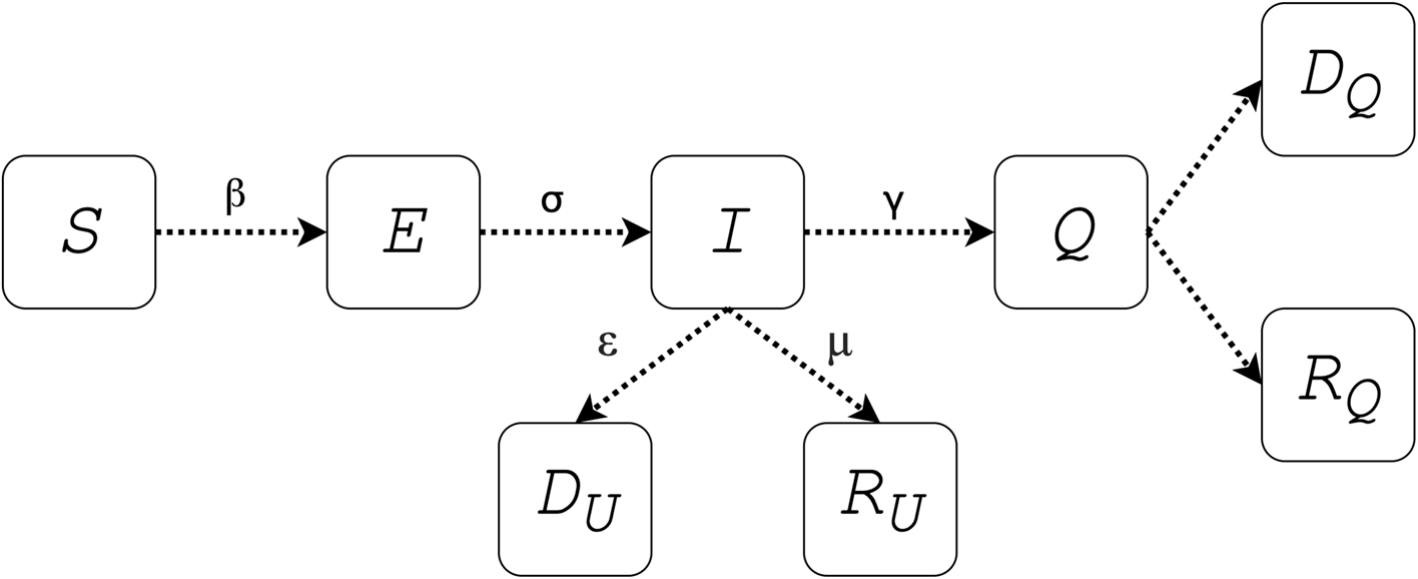
Epidemic spreading diagram for SEIQ model. *S*: susceptible; *E*: exposed; *I*: infective; $Q$: quarantined. Parameter $\beta$ indicates the infectious rate. Parameter $\sigma$ indicates the incubation rate with $\sigma =\frac{1}{TE}$ (incubation period). Parameter $\gamma$ indicates the quarantine rate with $\gamma =\frac{1}{TI}$ (infectious period).

SEIR model is a classic compartmental model that has been initially used to simulate the spread of flu. Some previous work employed the SEIR model to predict the trend for COVID-19, which assumed that the exposed individuals (who were infected but displays no symptoms) are not infective^2^. However, it has been reported that COVID-19 might be transmissible for exposed individuals^11^. Moreover, R compartment in the traditional SEIR model indicates recovered cases or more precisely removed cases, who were removed from the total population and lost their infective or susceptible properties. Unlike flu patients who recovered soon after treatment or untreated, there was no specialized treatment for COVID-19, and COVID-19 patients were usually quarantined quickly by health workers and lost their infective or susceptible properties. In this scenario, the counterpart of *R* compartment in the traditional SEIR model should be replaced quarantined compartment (*Q*). Therefore, the infectious period which was the time between state infection *(I*) and recovered (*R*) in the traditional SEIR model, corresponded to the time between state infection (*I*) and quarantined (*Q*) in the COVID-19 epidemic. Therefore, we replaced the recovered individuals *R* with the quarantined individuals *Q*, and the model became the SEIQ model. The quarantined individuals *Q* indicated the confirmed cases who were detected and centrally quarantined. The quarantined individuals *Q* became either recovered (*R*_*Q*_) or death (*D*_*Q*_) eventually. Meanwhile, some infected cases recovered or deceased without being detected and diagnosed. We defined those cases as undetected recovered (*R*_*u*_) and death (*D*_*u*_) cases. The epidemic spreading model for the SEIQ model is therefore illustrated in Fig. 1.

The transmission dynamics are governed by the following system of equations:

1 $$\begin{aligned}\frac{dS\left(t\right)}{dt} & =\frac{- S\left(t\right) I\left(t\right)}{N}\\ \frac{dE\left(t\right)}{dt}&=\frac{ S\left(t\right) I\left(t\right)}{N}- E\left(t\right)\\ \frac{dI\left(t\right)}{dt}& = E\left(t\right)- I\left(t\right)-\upvarepsilon I\left(t\right)-\upmu I\left(t\right)\\ \frac{dQ\left(t\right)}{dt}&= I\left(t\right)\end{aligned}$$

where $N=S\left(t\right)+E\left(t\right)+I\left(t\right)+Q\left(t\right)+{R}_{u}\left(\mathrm{t}\right)+ {D}_{u}(\mathrm{t})$ is the total population, which is assumed a constant.

Like the SEIR model, parameter $\beta$ indicates the infectious rate with $\beta =\frac{{R}_{t}}{TE}$ where ${R}_{t}$ is the dynamic effective reproduction number and $TE$ is the average duration of incubation; parameter $\sigma$ indicates the incubation rate with $\sigma =\frac{1}{TE}$. However, in our model, parameter $\gamma$ indicates the quarantine rate with $\gamma =\frac{1}{TI}$ (where $TI$ is the average duration of an infectious individual to be detected and quarantined). The parameter $TI$ reflects the timeliness of patient detection and admission and usually varies across different regions. Parameters ε and μ indicate the undetected recovered and death rate, respectively.

The basic reproduction number ${R}_{0}$ is the most important parameter to determine the intrinsic transmissibility of COVID-19, and it is defined as the average number of infections one infectious agent can generate over the course of the infectious period without any interventions. ${R}_{0}$ was assumed to be a constant or arbitrarily modified at specific points for forecasting in previous work^12,13^. However, in real-world scenarios, with the development of the epidemic, more and more interventions are often taken to control the spread, which gradually reduces ${R}_{0}$. In this work, the basic reproduction number ${R}_{0}$ is generalized to a dynamic value ${R}_{t}$, which is defined as the average number of secondary infectious cases generated by an infectious at time t. After the worldwide outbreak of COVID-19, many governments took considerable measures to contain the spread of the virus. In our preliminary analysis and some previous work^14^, the infectious rate $\beta$ was shown to decrease exponentially with time. As parameter $TE$ is constant, the effective reproduction number ${R}_{t}$ should follow a similar pattern as decreasing exponentially with time. Thus, we introduced time-dependent dynamics to the estimation of ${R}_{t}$ for better simulation of the real-world transmission,

2 $$\begin{array}{c}{R}_{t}={R}_{\infty }+\left({R}_{0}-{R}_{\infty }\right)\times {e}^{-t}\end{array}$$

where ${R}_{\infty }$ is the final reproduction number at the end of the pandemic and $\theta$ is the decrease ratio of the reproduction number, which is associated with the corresponding interventions. At the very beginning when $t=0$, ${R}_{t}={R}_{0}$, and it gradually reduces to ${R}_{\infty }$ as *t* increases. The epidemic is considered to be under control with ${R}_{t}<1$, and the reasonable range of ${R}_{\infty }$ was provided in some previous analysis of coronavirus^15^.

### Parameter constraints and optimization

The simulation and prediction of the D-SEIQ model require the determination of parameters ${R}_{0},{R}_{\infty }, TE, TI, \theta$. Although we incorporated machine learning techniques to help us to fit the reported data, the parameter range needs to be pre-set carefully and to conform to epidemiological rationality. For instance, Wu et al. applied an adjusted SEIR model to estimate ${R}_{0}$ (${R}_{0}=2.68$) in major cities of China by analyzing the number of cases exported from Wuhan internationally^6^. Some work concluded that the daily reproduction number varied between 2 and 7^16^. Therefore, we set a reasonable range for parameter ${R}_{0}\in [\mathrm{2,7}]$. Likewise, after reviewing the previous work on the analysis of COVID-19 [2, 11], we summarized the ranges for parameters in our model as Table 1. And, we set $TE>TI$ as an additional constraint. Therefore, the parameter optimization process is as follows:

**Table 1:** The constrained range for parameters with epidemic rationality.

Parameters	Reasonable ranges
$${R}_{0}$$	[2, 7]
$$TE$$	[3, 11]
$$TI$$	[1, 5]
$${R}_{\infty }$$	[0.05, 0.35]
$$\theta$$	[0.05, 0.45]

${R}_{0}$ denotes the basic reproduction number; $TE$ denotes the incubation period; $TI$ denotes the infectious period; ${R}_{\infty }$ denotes the final value of ${R}_{t}$; $\theta$ denotes the decrease ratio of ${R}_{t}$.

- Initialize the number of confirmed cases $Q$ at time $t=0$ according to the official report.
- Initialize the parameters ${R}_{0}, {R}_{\infty }, TE, TI, \theta$.
- Calculate the time-dependent effective reproduction number ${R}_{t}$.
- Solve ordinary differential equations in Eq. (1) to determine $E\left(t\right), I\left(t\right), Q(t)$.
- Set loss function as the sum of mean squared errors of daily and cumulative confirmed numbers, and then estimate the parameters ${R}_{0}, {R}_{\infty }, TE, TI, \theta$ based on grid search with dynamically adapted search steps to obtain the best D-SEIQ model at time $t$.

### Data processing

We obtained the updated data of the cumulative confirmed cases from the National Health Commission (NHC) of the People’s Republic of China. The newly confirmed cases were also collected on a daily basis. Considering that medical resources and interventions might vary in different regions, we fitted our model on the data from three different regions: (1) China excluding Hubei, (2) Hubei excluding Wuhan, and (3) Wuhan.

Moreover, we adjusted the number of newly confirmed cases in Wuhan between 12 and 14 February, due to the inclusion of clinically confirmed cases without coronavirus test. The clinically confirmed cases between 12 and 14 February were assumed to be suspicious cases in the last 7 days. Specifically, we redistributed the clinically confirmed cases according to the distribution of suspected cases over the past 7 days.

### Forecasting long-term trends of confirmed case numbers

Because China’s NHC publicly reported case numbers starting from 20 January, we set this date as the starting point of our training data. As of 10 March, the daily increased case numbers declined to single digits across most areas in China, we set this date as the ending point of our model.

We updated our models dynamically from the 7th day following the starting point (i.e., 27 January). In this article, we presented the prediction of our models at the time points of 1st to 5th week, namely 27 January, 4 February, 11 February, 18 February, and 25 February.

For example, the model for the first week (as of 27 January) used the data from 20 to 26 January for model construction and forecasted the daily increased and cumulative case numbers from 27 January to 10 March.

As of 27 April, the date on which the manuscript was finished, we used the same model to make a one-month prediction for the top five countries with worst outbreaks, including the United States, Italy, Spain, Germany, and France, to test the external validity of our models.

## Results

The simulation and prediction of our D-SEIQ models are illustrated from three different regions: China excluding Hubei, Hubei excluding Wuhan, and Wuhan.

### China excluding Hubei

**Figure 2:**
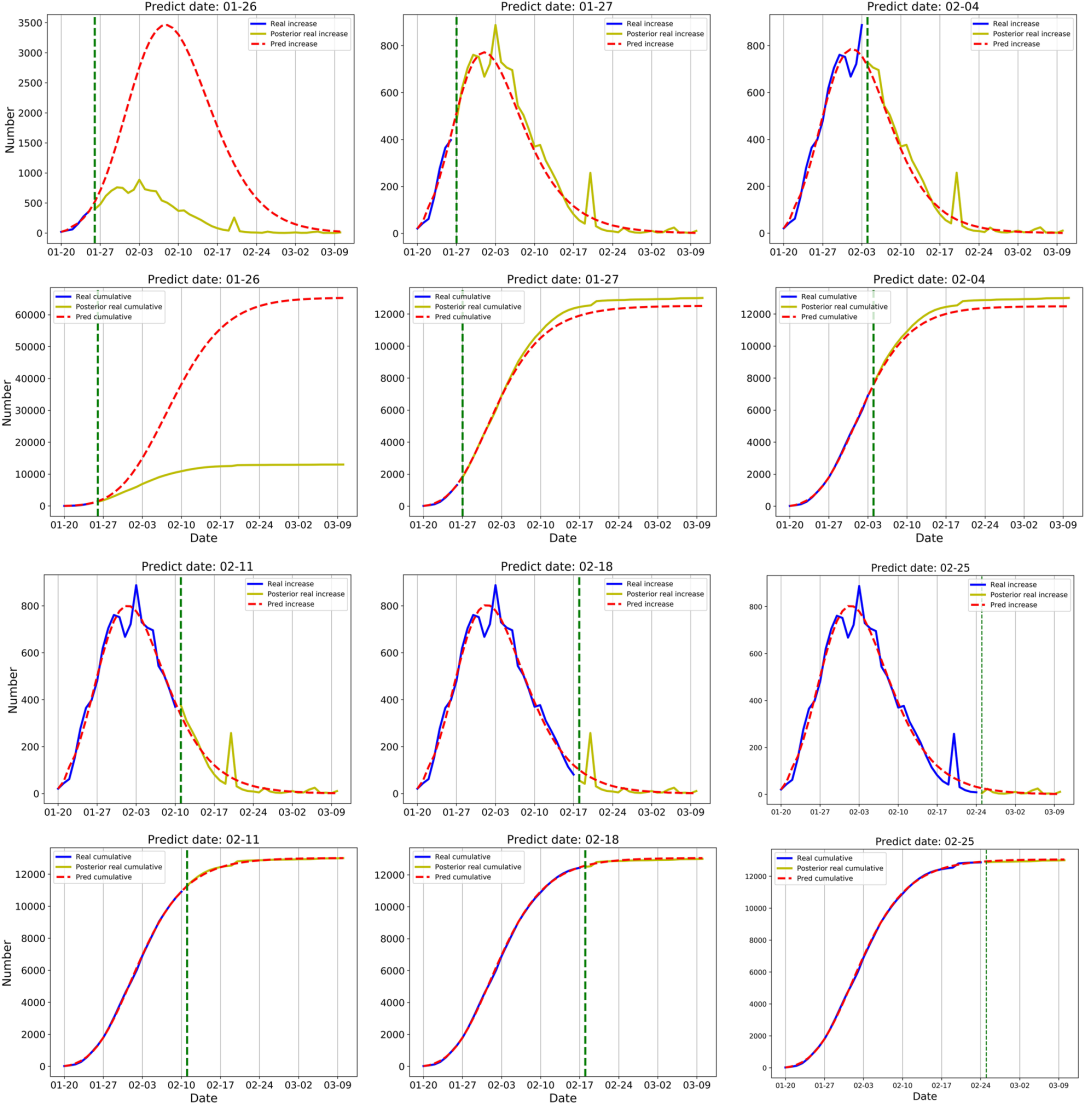
Simulation and prediction results of daily and cumulative confirmed cases in the region of China excluding Hubei. Data were shown on each figure with 6 different prediction dates. The results of daily confirmed cases are placed on 1st and 3rd rows and the results of cumulative confirmed cases are placed on 2nd and 4th rows. Green vertical line: the prediction date which separate training data and test data. Solid blue line: the real number of confirmed cases before prediction date, namely training data. Solid yellow line: the retrospective number of confirmed cases, namely test data. Red dotted line: the number predicted by the D-SEIQ model.

The D-SEIQ model with the prediction date of 26 January showed that the cumulative number would reach 65,282 (red dotted line in Fig. 2) on 10 March. In retrospect, our model greatly overestimated the development of the epidemic, possibly because at the early stage of the epidemic when intervention had not taken its effect, the number of cases increased sharply and did not show the potential decline of ${R}_{t}$. The overestimation also illustrated the effectiveness of the subsequent containment measures.

The D-SEIQ model trained on 27 January showed that the cumulative number would reach 12,506 on 10 March, and the daily number would reach the peak on 1 February. In retrospect, the prediction was quite close to the real scenario. The real cumulative number on 10 March was 13,005 which was only 3.8% different from the predicted value. Also, the outbreak peak predicted by our model is exactly the same as the actual date (around 1 February to 3 February). Therefore, in the region of China excluding Hubei, the D-SEIQ model is shown to successfully estimate the trend for up to 40 days, with one-week data after the first public report.

At the late stage of epidemic spread, the model is capable of fitting on previous data and also predicting the epidemic development. For example, on 11 February, we predicted the cumulative number was 13,006 at the endpoint while the true value is 13,005.

The parameters learned at the late stage could accurately reflect the intrinsic characteristics of COVID-19. Thus, the parameters on 25 February were used as the estimation of true values. In the region of China excluding Hubei, the basic reproduction number ${R}_{0}$ was estimated to be 6.3; the decrease ratio $\theta$ to be 0.2; the incubation period $TE$ to be 3 days, and the infectious period $TI$ to be 2 days. The effective reproduction number ${R}_{\infty }$ ultimately dropped to around 0.3.

### Hubei excluding Wuhan

The number of confirmed cases grew rapidly in the region of Hubei excluding Wuhan in the first week, which biased our model of 27 January to enormously overestimate the peak value. Our model predicted that the cumulative number would reach 65,763 by 10 March. On the other hand, the overestimation also indicates that, without containment, the epidemic would show explosive growth as the influence of containment measures remained unseen at the early stage of the epidemic.

**Figure 3:**
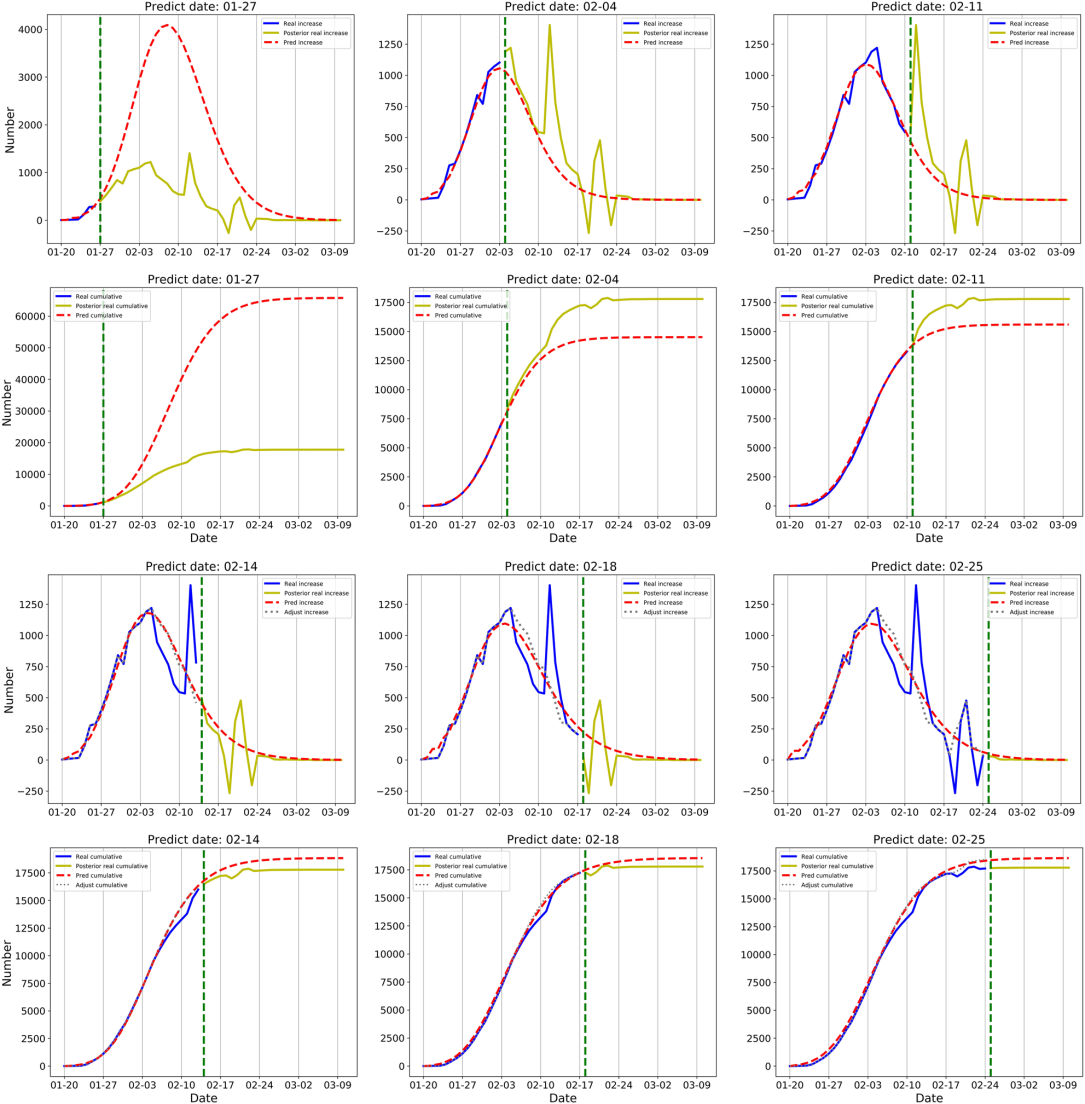
Simulation and prediction results of daily and cumulative confirmed cases in the region of Hubei excluding Wuhan. Data were shown on each figure with 6 different prediction dates. The results of daily confirmed cases are placed on 1st and 3rd rows and the results of cumulative confirmed cases are placed on 2nd and 4th rows. Green vertical line: the prediction date which separate training data and test data. Solid blue line: the real number of confirmed cases before prediction date, namely training data. Solid yellow line: the retrospective number of confirmed cases, namely test data. Red dotted line: the number predicted by the D-SEIQ model. Grey dashed line: the numbers after adjustment of the clinically confirmed cases.

After the clinically confirmed cases between 12 and 14 February were adjusted by redistribution, we re-trained our model with adjusted values (Fig. 3). The model on 14 February after adjustment showed that the cumulative number would reach 18,844 with an error of 6% compared with the real number.

Similarly, based on the model of the late stage of the epidemic (25 February), the transmission parameters of the virus were estimated as follows: the basic reproduction number ${R}_{0}$ was 6.3; the decrease ration $\theta$ was 0.15; the final reproduction number ${R}_{\infty }$ was 0.2; the incubation period $TE$ was 3 days; and the infectious period $TI$ was 2 days.

### Wuhan

**Figure 4:**
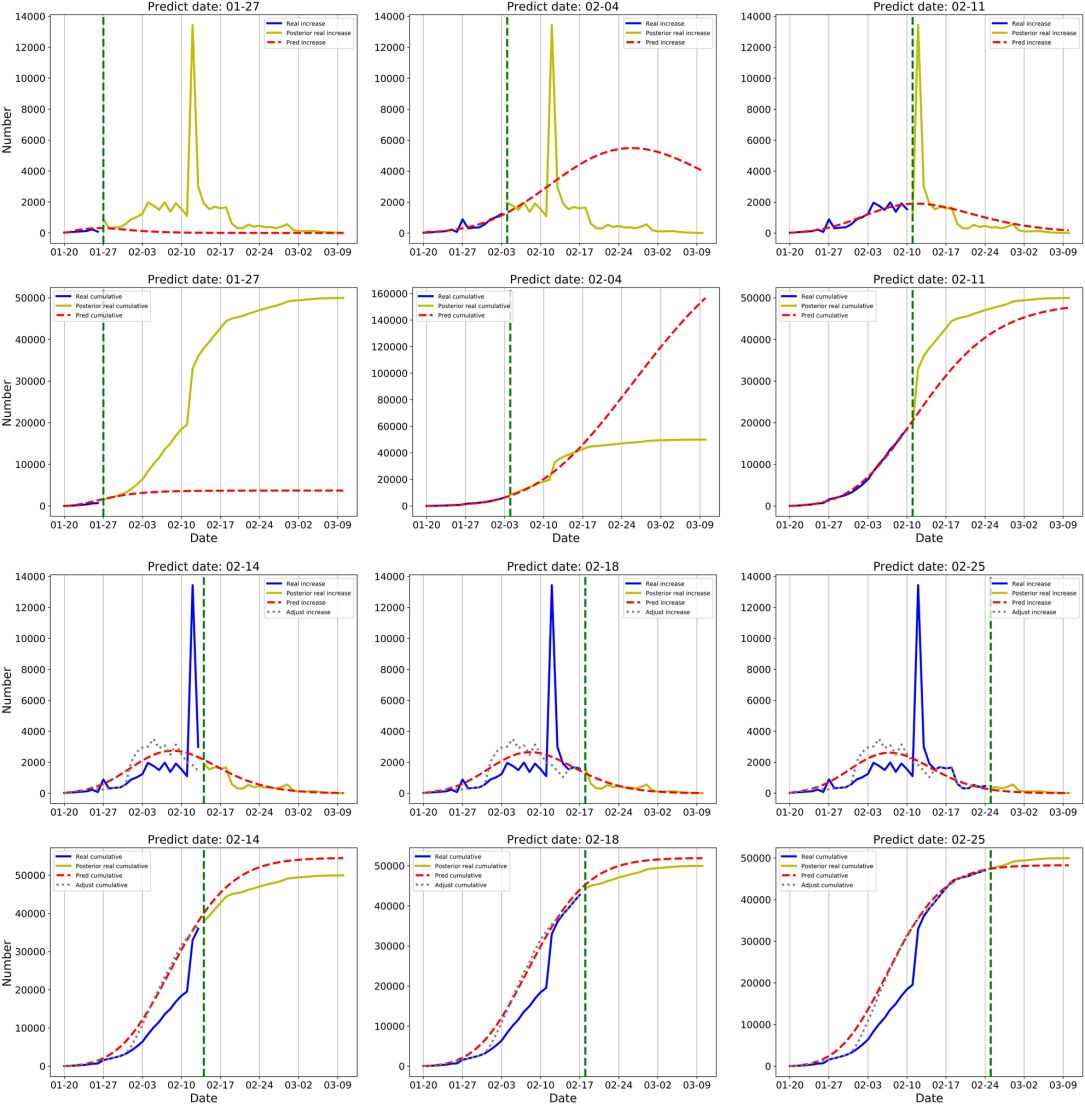
Simulation and prediction results of daily and cumulative confirmed cases in the region of Wuhan Data were shown on each figure with 6 different prediction dates. The results of daily confirmed cases are placed on 1st and 3rd rows and the results of cumulative confirmed cases are placed on 2nd and 4th rows. Green vertical line: the prediction date which separate training data and test data. Solid blue line: the real number of confirmed cases before prediction date, namely training data. Solid yellow line: the retrospective number of confirmed cases, namely test data. Red dotted line: the number predicted by the D-SEIQ model. Grey dashed line: the numbers after adjustment of the clinically confirmed cases.

In the early days of the epidemic outbreak in Wuhan, due to the deficiency of detection capabilities and limited medical resources, the reported numbers were far below the real incidences. During the first week, the daily increased numbers even showed a declining trend, and the D-SEIQ model of 27 January consequently underestimated the epidemic development. There was a large increase in clinically confirmed cases between 12 and 14 February. We adjusted the numbers on 14 February and the prediction showed that the cumulative number would reach 54,492 at the endpoint, with an error of 9% from the actual number of 49,980. On 18 February, the D-SEIQ model showed a convincing simulation of the overall trend, and the overall predicted curve indeed fitted the adjusted values quite well (grey dashed line in Fig. 4).

The estimated parameters of the COVID-19 transmission were as follows: the basic reproduction number ${R}_{0}$ was estimated to be 4.63; the decrease ratio $\theta$ was 0.1; the final reproduction number ${R}_{\infty }$ was 0.15; the incubation period $TE$ was 3 days; and the infectious period $TI$ was 2.5 days.

### **Analysis of reproduction number** R_t_

**Figure 5:**
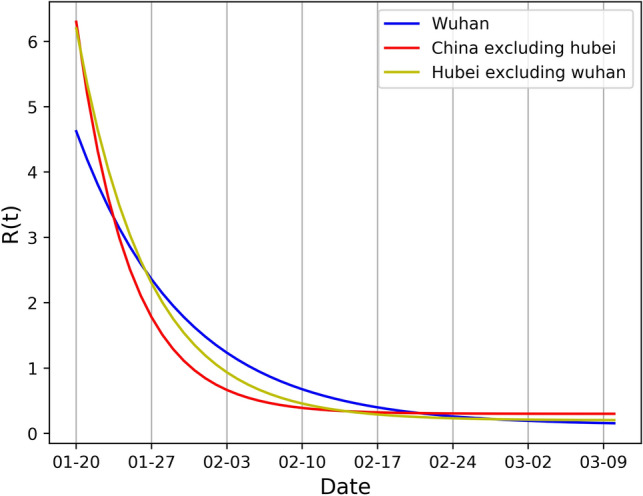
The estimated dynamic effective reproduction number ${R}_{t}$ in three different regions of China. Blue line: Wuhan city. Yellow line: Hubei province excluding Wuhan city. Red line: China excluding Hubei province.

We further analyzed the reproduction number ${R}_{t}$ by our D-SEIQ models. We used the ${R}_{t}$ learned at the late stage of the simulation. We plotted the ${R}_{t}$ curve from 20 January to 10 March as Fig. 5 to compare the reproduction numbers in three different regions. At the initial time, ${R}_{0}$ was 6.3 in China excluding Hubei and Hubei excluding Wuhan, both of which were larger than that in Wuhan with ${R}_{0}=4.63$. However, the decrease ratio $\theta$ for ${R}_{t}$ was largest in China excluding Hubei (0.20), followed by Hubei excluding Wuhan and then Wuhan. Therefore, ${R}_{t}$ in China excluding Hubei dropped below 1 the earliest, meaning that COVID-19 was under control in other provinces sooner than Hubei province. The final ${R}_{\infty }$ of three different regions all approached zero, demonstrating a great achievement in epidemic containment and interventions.

### Forecasting long-term trends of COVID-19 for countries outside of China

As of 27 April when this manuscript was finished and most countries have reached the late stage of the first epidemic wave, we forecasted a one-month trend of COVID-19 for the top 5 countries with worst outbreaks using the proposed D-SEIQ model, namely the US, Spain, Italy, France, and Germany. The one-month ahead predicted cumulative reported numbers of cases were fairly close to the real numbers for all the five countries except the US (Supplementary Fig. 1). The differences between the predicted and the real numbers were − 16.0% (the US), − 7.5% (Spain), − 8.5% (Italy), − 7.7% (France), and − 0.2% (Germany), respectively.

## Discussion

We proposed a new model named D-SEIQ, which applies appropriate modifications of the SEIR model and combines with parameter optimization of machine learning. We evaluated our model on officially reported data from three different regions in China, and the results proved the effectiveness of our model in terms of simulating and predicting the trend of COVID-19 outbreak and regional spread. Especially, in China excluding Hubei area within 7 days after the first public report, our model successfully and accurately predicted the long trend up to 40 days and the exact date of the outbreak peak.

Traditional epidemic transmission models like SEIR need an accurate estimation of model parameters such as basic reproduction number, incubation period, and infectious period through epidemiological investigation. However, in terms of a new epidemic, due to the rapid outbreak, insufficient sample size, and the deviation of investigated data from the ground-truth, the traditional epidemic transmission models usually poorly fit to the data. In practice, researchers often made various assumptions for calculation or even used relevant parameters of other viruses as substitutions. For example, Wu et al. adopted serial interval estimates for SARS as substitutions and estimated that 75,815 individuals were infected in Wuhan as of 25 January^6^, which significantly overestimated the figure. On the other hand, machine learning methods, such as logistic regression models, were subject to overfitting problems^17^, which means they could fit the training data well but fail to predict on unseen data. The accountable reasons include the limited epidemic rationality of the models and the insufficiency of data and salient features. Deep neural networks like long short-term memory (LSTM) were proven to be incapable of predicting the long-term trends and the outbreak peak^18^.

Our model takes advantage of both epidemic and machine learning models, which combine the explainability of the epidemic model with the data-fitting ability of machine learning. In the process of machine learning, we set the parameters within a reasonable range, and exploit mutual constraints between the parameters.

Meanwhile, we innovatively introduced dynamic ${R}_{t}$, which can reflect the time-dependent influence of intervention measures on basic reproduction number. Overall, our approach could more accurately simulate the real-world scenario of the COVID-19 spread, thus making better prediction.

Furthermore, the parameters learned by our D-SEIQ model could provide some insights into the assessment of the prevention and containment measures on COVID-19. Firstly, the basic reproduction number was relatively large (4–6), which was larger than SARS-COV with ${R}_{0}$ ranging from 1.6 to 3.7^15,19,20^. Without strong and effective intervention measures including city lockdown, travel containment, mask-wearing, quarantine, and screening, it could lead to catastrophic consequences to society. The final reproduction number of different areas of China gradually dropped to around 0.2, illustrating the considerable effect and the significant importance of interventions from governments or the public. Secondly, the decrease ratio of ${R}_{t}$ was slower in Wuhan which indicates the shortage of medical resources and delayed patient admission in Wuhan. This conclusion is also supported by the estimated infectious period ($TI$), which has a larger value in Wuhan than other regions of China. Moreover, our model obtained the same incubation period ($TE$) with 3 days across three regions, which was consistent with that from the Chinese CDC official report^11^.

The D-SEIQ model is applicable only when the following conditions are satisfied: adequate medical capacities, consistency of containment measures and ascertainment criteria, and timely case detection and reporting. This explained the reason why our model performed better in China excluding Hubei region. Therefore, caution needs to be taken when applying our model to other countries. The detection and reporting were not timely in some countries like the United States at the early phase, and subsequent containment measures were introduced and lift at different time points, which might influence the prediction results. Another limitation was that our model can only predict the trend of a single epidemic wave. Recently, China as well as some other countries have seen a second wave of the epidemic due to imported cases or relaxed containments. Mathematical models are currently not available to predict the possibility of the second wave.

## Conclusion

We have proposed a new approach for forecasting the COVID-19 long-term trend. The model has accurately predicted the long-term trend of the epidemic in China, and the parameters learned from the model suggested the effectiveness of the intervention measures that have been conducted in China, which can help us analyze and fight against the new epidemic.

## Supplementary information

Supplementary Information.

## Data Availability

The data sets used in this study are freely available to public on the webpage: https://ourworldindata.org/coronavirus. The codes and processed data for different regions of China are available on GitHub: https://github.com/jichaosun001/covid_forecast.git.

## References

[1] WHO. Naming the coronavirus disease (COVID-19) and the virus that causes it. https://www.who.int/emergencies/diseases/novel-coronavirus-2019/technical-guidance/naming-the-coronavirus-disease-(covid-2019)-and-the-virus-that-causes-it (2020).

[2] Zhu N (2020). A novel coronavirus from patients with pneumonia in China, 2019. N. Engl. J. Med..

[3] CSSE at Johns Hopkins University. *Coronavirus COVID-19 Global Cases by the Center for Systems Science and Engineering (CSSE) at Johns Hopkins University (JHU).* https://www.arcgis.com/apps/opsdashboard/index.html#/bda7594740fd40299423467b48e9ecf6 (2020).

[4] WHO. WHO Director-General's opening remarks at the media briefing on COVID-19. Available at https://www.who.int/director-general/speeches/detail/who-director-general-s-opening-remarks-at-the-media-briefing-on-covid-19. 11 Mar 2020 (2020).

[5] Zhou, X. *et al.* Forecasting the Worldwide Spread of COVID-19 based on Logistic Model and SEIR Model. medRxiv, 2020.2003.2026.20044289. 10.1101/2020.03.26.20044289 (2020).

[6] Wu JT, Leung K, Leung GM (2020). Nowcasting and forecasting the potential domestic and international spread of the 2019-nCoV outbreak originating in Wuhan, China: A modelling study. Lancet.

[7] Tátrai, D. & Várallyay, Z. COVID-19 epidemic outcome predictions based on logistic fitting and estimation of its reliability. arXiv e-prints, arXiv:2003.14160 (2020).

[8] Roosa K (2020). Real-time forecasts of the COVID-19 epidemic in China from February 5th to February 24th, 2020. Infect. Dis. Model..

[9] Jia, L., Li, K., Jiang, Y., Guo, X. & Zhao, T. Prediction and analysis of Coronavirus Disease 2019. arXiv e-prints, arXiv:2003.05447 (2020).

[10] Yang Z (2020). Modified SEIR and AI prediction of the epidemics trend of COVID-19 in China under public health interventions. J. Thorac. Dis..

[11] Guan W-J (2020). Clinical characteristics of Coronavirus Disease 2019 in China. N. Engl. J. Med..

[12] Read, J. M., Bridgen, J. R., Cummings, D. A., Ho, A. & Jewell, C. P. Novel coronavirus 2019-nCoV: Early estimation of epidemiological parameters and epidemic predictions. medRxiv, 2020.2001.2023.20018549. 10.1101/2020.01.23.20018549 (2020).

[13] Gupta, R., Pandey, G., Chaudhary, P. & Pal, S. K. SEIR and Regression Model based COVID-19 outbreak predictions in India. medRxiv, 2020.2004.2001.20049825. 10.1101/2020.04.01.20049825 (2020).

[14] Chen, B. *et al.* Visual Data Analysis and Simulation Prediction for COVID-19. arXiv e-prints, arXiv:2002.07096 (2020).

[15] Riley S (2003). Transmission dynamics of the etiological agent of SARS in Hong Kong: Impact of Public Health Interventions. Science.

[16] Kucharski AJ (2020). Early dynamics of transmission and control of COVID-19: A mathematical modelling study. Lancet Infect. Dis..

[17] Batista, M. Estimation of the final size of the COVID-19 epidemic. medRxiv, 2020.2002.2016.20023606. 10.1101/2020.02.16.20023606 (2020).

[18] Zheng N (2020). Predicting COVID-19 in China using hybrid AI model. IEEE Trans. Cybern..

[19] Chowell G (2004). Model parameters and outbreak control for SARS. Emerg. Infect. Dis..

[20] Lloyd-Smith JO, Schreiber SJ, Kopp PE, Getz WM (2005). Superspreading and the effect of individual variation on disease emergence. Nature.

