# Supplementary material for: Forecasting the long-term trend of COVID-19 epidemic using a dynamic model
Source: Sci Rep. 2020 Dec 3;10:21122. doi: 10.1038/s41598-020-78084-w (PMC7713358; doi:10.1038/s41598-020-78084-w)

**Forecasting the long-term trend of COVID-19 epidemic using a dynamic model**

**Running title**

Long-term trend of COVID-19 forecasting model

**Authors**

Jichao Sun,^†^ Xi Chen,^†^ Ziheng Zhang,^†^ Shengzhang Lai, Bo Zhao, Hualuo Liu, Shuojia Wang, Wenjing Huan, Ruihui Zhao, Man Tat Alexander Ng*, Yefeng Zheng*

^†^ Jichao Sun, Xi Chen, and Ziheng Zhang contributed equally to this work as co-first authors.

Jarvis Lab, Department of Medicine and Healthcare, Tencent Technology (Shenzhen) Company (J Sun PhD, X Chen PhD, Z Zhang MSc, S Lai MSc, B Zhao PhD, H Liu MSc, S Wang PhD, W Huan MSc, R Zhao MSc, M-T-A Ng MSc, Y Zheng PhD)

***Correspondence to**

Dr Yefeng Zheng, Director of Jarvis Lab, Department of Medicine and Healthcare, Tencent Technology (Shenzhen) Company, Shenzhen 518000, China; Email: [yefengzheng@tencent.com](mailto:yefengzheng@tencent.com)

Or

Mr Man Tat Alexander Ng, General Manager of Department of Medicine and Healthcare, Tencent Technology (Shenzhen) Company, Shenzhen 518000, China; Email: [alexanderng@tencent.com](mailto:alexanderng@tencent.com)

Supplementary Figure 1. Forecasting long-term trends of COVID-19 for countries outside of China


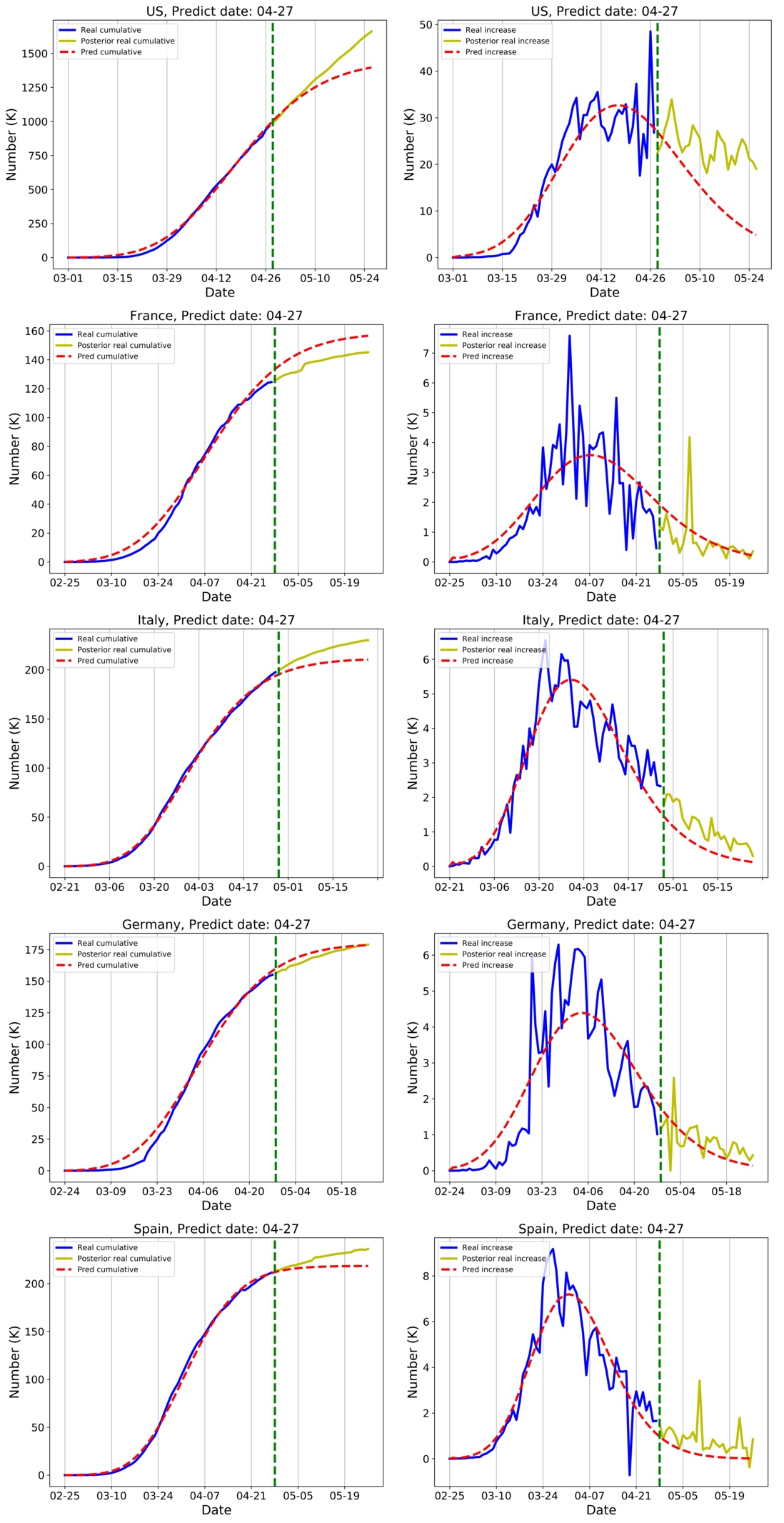

Supplement: Supplementary file 1 — Supplementary Information. [file 41598_2020_78084_MOESM1_ESM.docx]
